# Concussion-Related Decision-Making by Certified Athletic Trainers: Implications for Concussion Prevention and Care

**DOI:** 10.3390/ijerph21010082

**Published:** 2024-01-11

**Authors:** Melissa K. Kossman, Zachary Yukio Kerr, J. D. DeFreese, Kristen L. Kucera, Meredith A. Petschauer, Kurt M. Ribisl, Johna K. Register-Mihalik

**Affiliations:** 1 School of Health Professions, University of Southern Mississippi, Hattiesburg, MS 39406, USA; 2 Sports Medicine and Community Health Research Lab, University of Southern Mississippi, Hattiesburg, MS 39406, USA; 3 Department of Exercise and Sport Science, University of North Carolina at Chapel Hill, Chapel Hill, NC 27599, USA; zkerr@email.unc.edu (Z.Y.K.); kkucera@email.unc.edu (K.L.K.);; 4 Human Movement Science Curriculum, University of North Carolina at Chapel Hill, Chapel Hill, NC 27599, USA; 5 Matthew Gfeller Center, University of North Carolina at Chapel Hill, Chapel Hill, NC 27599, USA; 6 Injury Prevention Research Center, University of North Carolina at Chapel Hill, Chapel Hill, NC 27599, USA; 7 Center for Study of Retired Athletes, University of North Carolina at Chapel Hill, Chapel Hill, NC 27599, USA; 8 National Center for Catastrophic Sport Injury Research, University of North Carolina at Chapel Hill, Chapel Hill, NC 27599, USA; 9 Department of Health Behavior, University of North Carolina at Chapel Hill, Chapel Hill, NC 27599, USA; 10 STAR Heel Performance Laboratory, University of North Carolina at Chapel Hill, Chapel Hill, NC 27599, USA

**Keywords:** athletic training education, communication, concussion prevention, integrated behavior model

## Abstract

Concussions are a common sport-related injury that require appropriate initial care. Athletic trainers, often a primary source of healthcare for student-athletes, are key individuals involved in initial concussion diagnostic and management decisions. Challenges exist within the athletic environment that may hinder the consistency, efficacy, and/or effectiveness of concussion-related decision-making by athletic trainers, thereby impacting secondary concussion prevention and patient health. The purpose of this study was to identify factors that impact the intentions of athletic trainers to make appropriate concussion-related decisions under various circumstances. Overall, 1029 participants completed a survey examining educational precursors (quantity and quality of healthcare communication educational focus), demographic precursors (age, gender, educational degree, and employment setting), theory-based mediators (attitudes, perceived norms, and personal agency), and external mediators (knowledge, salience, and communication/collaboration practices) on appropriate concussion-related decision-making intentions. Data were analyzed using a two-step structural equation modeling approach. Quality of healthcare communication educational focus indirectly impacted appropriate concussion-related decision-making intentions via perceived behavioral control and communication/collaboration practices. Additionally, several factors impacted intentions to make appropriate concussion-related decisions directly including employment setting, self-efficacy, and general attitudes towards decision-making and concussions. Concussion prevention is aided by the initial and appropriate action taken by a healthcare professional to reduce immediate consequences; however, this action may be influenced by stakeholder relationships. These influential factors of decision-making may place athletes at further injury risk and negatively impact overall athlete health. As such, a sound theoretical framework incorporating the complexity of factors that may influence decision-making is needed.

## 1. Introduction

Concussions are a relatively common, but complex injury that occur at a high rate [1,2]. These injuries are due to trauma to the head and/or body and result in various neurological deficits, including cognitive, balance, and symptom changes [3]. As such, concussive injuries impact athletic performance, school performance, and social interactions [4,5,6]. Therefore, appropriate injury management by trained healthcare professionals is imperative to reduce additional burden. Athletic trainers (ATs) are recognized allied health providers with a particularly unique position. Given the breadth and depth of their education, they are situated to take a leadership role in injury prevention, emergency care, clinical diagnosis, therapeutic intervention, and rehabilitation of concussions. This leadership role is in part due to their ability to witness the injury and have continual interaction with concussed athletes, but also their educational competency specific to concussive injuries. In particular, they can aid in the primary (preventing concussions from ever happening), secondary (reducing the immediate consequences), and tertiary (reducing the long-term burden) prevention of concussions within various sports settings [7]. The presence of ATs, combined with appropriate decision-making, has been shown to not only improve concussion reporting, but also post-injury concussion management [8].

The decision to withhold an athlete from participation due to injury can pose a challenge to healthcare professionals due to conflicting expectations and experiences [9,10]. Previous literature supports that nearly two-thirds of sports medicine clinicians have felt pressure to prematurely return a concussed athlete to the playing field [11]. These pressures have been particularly evident in recent media and news outlets highlighting negative patient outcomes (i.e., athlete death) resulting from a lack of appropriate care [12]. Preliminary data conducted by the research team suggests the struggle of making appropriate concussion-related decisions is due to the lack of effective communication and collaboration [blinded for peer review]. The extent to which communication (unidirectional delivery of information) and collaboration (multidirectional shared expertise), as well as other factors, affect an AT’s ability to make appropriate decisions is unclear and is therefore the primary focus of this investigation. ATs may be less likely to act upon an appropriate decision about concussions during stressful situations when their intentions are met with competing influence. Inappropriate decisions place athletes at further risk of injury and can negatively impact their overall health. As such, a sound theoretical framework incorporating the complexity of factors that may influence decision-making is needed.

The Integrated Behavior Model (IBM) provided deductive context to this study using intention (to make appropriate concussion-related decisions) as the outcome of interest as it is a strong proxy to actual behavior [13]. Within the IBM, there are several proposed immediate theory-based constructs that influence that intention including (1) attitudes, (2) perceived norms, and (3) personal agency. Additional immediate external factors are proposed to influence intention including: (1) knowledge, (2) salience, (3) communication/collaboration, (4) environmental constraints, and (5) habit [14]. Within athletic training specifically, some factors are difficult to identify and modify due to the nature of sport (i.e., environmental constraints and habit); therefore, they are not a focus in this study.

There is currently a need for pragmatic and evidence-based interventions to improve AT decision-making and confidence concerning concussion to decrease subsequent harm following concussion in student-athletes and to promote long-term health and quality of life. However, to date, no studies have examined the factors that could be targeted in such an intervention to improve job effectiveness; rather, studies have focused on AT health [15,16], which may in turn affect job effectiveness. The purpose of this study was to identify factors which impact ATs intentions to make appropriate initial decisions to remove a concussed athlete from participation using a deductive approach. We hypothesized that four types of factors would impact these intentions including (1) educational precursors (quality and quantity of educational focus on healthcare communication), (2) demographic precursors (age, gender, educational degree, employment setting), (3) intermediate theory-based constructs (attitudes, perceived norms, personal agency), and (4) intermediate external factors (knowledge, salience, communication/collaboration practices). We used a deductive approach to help evaluate the appropriateness of IBM to describe the phenomenon of appropriate concussion-related decision-making in sport.

## 2. Materials and Methods

### 2.1. Study Sample

The research team obtained a list of 8974 ATs working in the high school or collegiate setting from the National Athletic Trainers’ Association (NATA). Of those initially surveyed, a total of 1369 (15.3% response rate) responded. The research team obtained approval from the [blinded for review] Institutional Review Board prior to initiation of survey distribution. All surveys were delivered to participants via email using the web-based program, Qualtrics (Qualtrics, LLC, Provo, UT, USA). Survey distribution consisted of three waves using random sampling without replacement. Two reminder emails were sent every two weeks with each respective wave for a total open period of 6 weeks per wave. Questions were built into the Qualtrics survey to verify inclusion criteria described earlier. Individuals determined to be ineligible by those questions were automatically prohibited from completing the survey.

Upon verification of inclusion criteria (registered with the NATA and certified in or after 2012), 1029 individuals qualified for survey completion with 413 (40.1%) individuals completing the survey entirely (age = 26.0 ± 3.7 years). Participants with 60% or more of completed data (*n* = 569) were included in the analysis. Those with less were excluded from further analyses (*n* = 460). Certification in or after 2012 was specifically used as criteria based upon changes in educational competencies that were implemented in that year. Full participant demographics are available in Table 1. The sample was primarily Caucasian (*n* = 464, 90.6%) and female (*n* = 377, 73.6%), consistent with the larger population [17], with diverse geographical representation. In addition, the majority of participants held a master’s (*n* = 313, 61.1%) as their highest earned degree, had an average years of experience of 3.1 years (±1.8), served as the head AT (*n* = 155, 30.5%), and worked under an athletic department reporting structure (*n* = 372, 73.8%). The ATs within this study worked in high school (*n* = 242, 43.0%) and collegiate (*n* = 271, 48.1%) settings with a diverse representation of non-contact, contact, and collision sport experience.

### 2.2. Measures

A cross-sectional survey examined factors associated with the intention to make concussion-related decisions by developing measures related to precursor variables (quality and quantity), intermediate variables (theory-based: attitudes, perceived norms, personal agency; peripheral factors: knowledge, salience, communication/collaboration), and demographic mediators (age, gender, educational degree held, and employment setting). Additionally, the measures were specifically designed according to IBM recommendations [13]. Single-item measures have demonstrated similar predictive validity to multi-item scales in studies of survey development [18,19]. The designed survey instrument was pilot tested with 15 ATs for face and content validity. Operational definitions for each construct are outlined below. Questions per survey construct can be found in Appendix A.

Quality of healthcare communication educational focus was assessed using two summed items (7-point Likert scale; range 2–14), with a higher score indicating more positive perception of educational quality.

Quantity of healthcare communication educational focus was assessed using three summed continuous standardized variables (range 0–∞), with a higher score indicating greater quantity of educational focus related to healthcare communication.

Attitudes toward making an appropriate concussion-related removal decision were measured by asking participants about the affective result of the behavior (experiential) and evaluation of performing the behavior (instrumental). Fourteen items were rated on a 7-point semantic differential scale and summed for a total possible continuous range of 14–98 where a higher score indicated more positive attitudes toward concussion-related removal decisions.

Perceived norms of what most ATs would do and approve of regarding the decision to withhold a concussed athlete were measured via 2 bipolar items for a possible range of −6 to 6 with higher scores indicating more positive perceptions of other AT behaviors.

Personal agency was comprised of self-efficacy and perceived behavioral control. All 5 items were ranked on a 7-point Likert scale and summed for a possible range of 5 to 35 with higher scores indicating stronger personal agency to make concussion-related decisions.

Knowledge contained 25 symptoms and consequences of concussion ranked on a scale of 1 (definitely not a symptom/consequence) to 4 (definitely is a symptom/consequence) for a total possible range of 25 to 100 with higher scores indicating better concussion knowledge.

Salience was measured by a single item asking about the level of priority placed upon concussion-related decision-making on a scale from 0 to 100 with a higher score indicating a higher priority.

Communication and collaboration experiences encompassed three dichotomized items regarding perceived preparation for decision-making and the number of times communicating/collaborating about concussion-related decisions. The three dichotomized items were then summed for a possible range of 0 to 3 with a higher score indicating more experience and perceived preparation for communicating/collaborating relative to concussion.

Intention to make appropriate concussion-related decisions was measured by summing 14 7-point bipolar scaled items regarding location, event type/time in season, and circumstance (i.e., pressure) for a possible range of −45 to 45 with higher scores indicating stronger intentions to make the appropriate medical decision according to best-practice guidelines for concussion.

### 2.3. Statistical Analysis

The initial step of data analysis included calculating descriptive statistics for all constructs via SAS (Version 9.4, Cary, NC, USA). As data were skewed left toward more positive values within constructs, medians and interquartile ranges were reported. Consistent with best practice, a two-step deductive structural equation modeling (SEM) approach [20] examined measurement of constructs and relationships among variables via MPlus (Version 8, Muthén & Muthén, Los Angeles, CA, USA) with an a priori alpha level of 0.05.

A confirmatory factor analysis (CFA) examined the relationship between measured variables (i.e., removing someone for a concussion is easy/hard) and their associated construct or latent variable (i.e., attitudes). We started by examining each construct in general with all items, followed by breaking constructs according to the IBM (i.e., attitudes became experiential attitudes and instrumental attitudes). Theory adaptation allowed for better measurement model fit (i.e., attitudes became general attitudes versus attitudes of complexity as opposed to experiential and instrumental attitudes).

Next, a structural path analysis examined the relationship between educational (quantity and quality of educational focus on decision-making) and demographic (age, gender, setting of employment, and educational degree held) precursors, and intermediate variables (attitudes toward concussion and decision-making, perceived norms towards concussion-related decision-making, personal agency regarding concussion-related decision-making, knowledge of concussions, salience of concussions, and communication/collaboration experiences) on the intention to make appropriate concussion-related decisions defined by best practices. The parameters in the structural equation model were estimated using the maximum likelihood method [21]. Additionally, for cases with greater than 60 percent completion (*n* = 569), mean imputation occurred for all primary variables of interest (affecting 27.6% of participants) [22]. Constructs for the path analysis did not significantly differ from individuals with full survey completion (*n* = 413).

**Figure 1:**
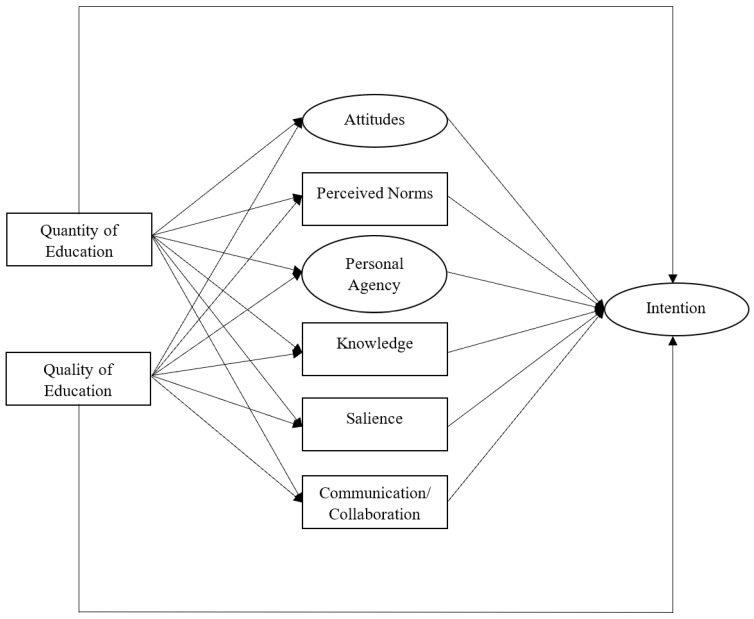
Proposed certified athletic trainer model for path analysis.

The order of variable entry in the model was based on the hypothesized path diagram (Figure 1). Specifically, attitudes, perceived norms, personal agency, communication/collaboration, salience, and knowledge were entered first as they were expected to be the most proximal predictors of intention. The effects of potential precursors (educational and demographic) on intention were expected to be mediated by the primary IBM constructs (attitudes, perceived norms, personal agency) as well as the external factors (communication/collaboration, salience, knowledge) [23]. The model was evaluated based upon commonly accepted fit values including root-mean-square error of approximation (RMSEA; <0.10 = acceptable, <0.05 = good) and the comparative fit index (CFI; >0.90 = acceptable, >0.95 = good) [24].

## 3. Results

Descriptive statistics were computed for all model constructs (Table 2). Overall, for educational precursors, participants perceived a diverse range of time spent covering healthcare communication as a topic (Quantity: Median = 33.0, IQR = 20.0–60.0); however, the time that was spent was perceived as somewhat beneficial (Quality: Median = 10.0, IQR = 8.0–12.0).

Regarding theoretical factors, patients had positive attitudes towards concussions and decision-making (Attitudes: Median = 85.0, IQR = 79.0–90.0) with the lower values driven by decisional complexity, as opposed to disagreement that concussions can have negative outcomes due to care. Perceived norms were high indicating they felt ATs tend to follow best practices (Median = 3.0, IQR = 2.5–3.0). Lastly, personal agency was strong (Median = 29.0; IQR = 27.0–32.0) with the lower scores relating to perceived behavioral control, or their influence over removing a concussed individual from play as opposed to their training to do so (self-efficacy).

Other intermediate factors indicated moderate to strong communication and collaboration practices (Median = 3.0, IQR = 2.0–3.0), moderate to high knowledge of concussion (Median = 82.0, IQR = 79.0–85.0), and highly skewed salience towards concussion being a worthy sports medicine concern (Median = 100.0, IQR = 100.0–100.0). Intentions were high across the sample indicating a desire and willingness to make the best decision (Median = 39.0, IQR = 37.0–41.0) with the greatest levels of diversity arising from pressure-induced decisions by sport stakeholders (ex: coaches).

### 3.1. Confirmatory Factor Analyses

The CFA indicated acceptable to good fit (RMSEA = 0.05–0.09; CFI = 0.95–0.99) of the theoretical constructs measured. Observed variables all had significant pattern coefficients indicating measured variables were significantly associated with their corresponding latent construct. The latent attitudes variable (with all observed variables summed) had poor model fit and therefore was split into two latent variables (general attitudes, complexity) with acceptable fit for use in the tested model. In addition, personal agency was split into two latent variables (perceived behavioral control, self-efficacy) and intentions were split into three latent variables (pressure, setting, situation) all with acceptable–good levels of model fit (RMSEA = 0.05, CFI = 0.99 and RMSEA = 0.08, CFI = 0.99, respectively). The seven latent variables with acceptable-good levels of model fit were used for testing in the structural model below (general attitudes, complexity, perceived behavioral control, self-efficacy, intentions despite pressure, intentions despite setting, and intentions despite situation).

### 3.2. Overall Model Fit

**Figure 2:**
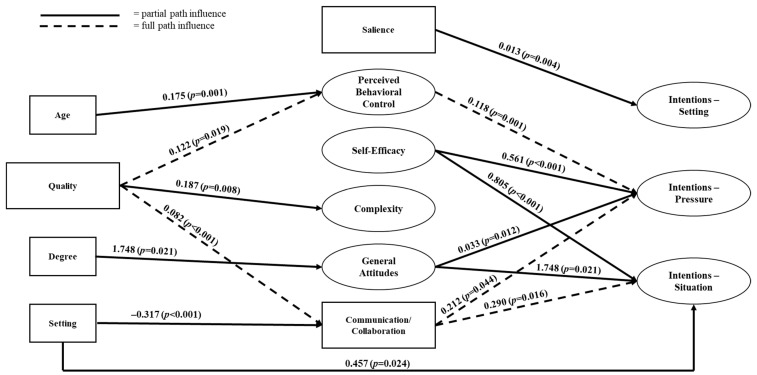
Certified Athletic Trainer Path Analysis Final Model. Model Fit Indices: RMSEA = 0.14, 95%CI: 0.13–0.16; CFI = 0.70. RMSEA, root mean square error of approximation; CFI, comparative fit index.

To test the proposed structural model (Figure 1), the educational and demographic precursors and external factors were added to the latent factors identified by the CFA (general attitudes, attitude complexity, perceived behavioral control, self-efficacy) based on the hypotheses described above. The structural model demonstrated poor fit to the data (RMSEA = 0.14, 95%CI: 0.13–0.16; CFI = 0.70) with modification indices suggesting many complex and overlapping influences [24]. Quantity, perceived norms and knowledge were removed from the model due to insignificant proximal and distal effects that caused worsened fit to the overall model. The remaining significant effects of quality, general attitudes, complexity, perceived behavioral control, self-efficacy, salience, and communication/collaboration practices are displayed in Figure 2. Educational quality had indirect effects on intentions related to pressure and the situation in which decision-making occurred. The specific demographic factors of age and degree level significantly impacted theoretical mediators while employment setting affected mediators and intentions dependent upon the situation.

### 3.3. Partial Path Predictors in Structural Model

The quality of educational focus on decision-making significantly influenced how complex individuals perceived concussion-related decision-making to be (β = 0.187, *p* = 0.008). In terms of intermediate variables, self-efficacy (β = 0.561, *p* < 0.001) and general attitudes (β = 0.033, *p* = 0.012) significantly influenced the intentions to make appropriate concussion-related decisions despite pressure (Table 3). These constructs also significantly influenced the intentions to make concussion-related decisions regardless of situation (self-efficacy: β = 0.805, *p* < 0.001; general attitudes: β = 0.047, *p* = 0.002; Table 3). Lastly, several demographic covariates significantly affected the intermediate variables including age on perceived behavioral control (β = 0.175, *p* = 0.001), educational degree on general attitudes (β = 1.748, *p* = 0.021), and employment setting on both communication/collaboration (β = −0.317, *p* < 0.001) and intentions regardless of setting (β = 0.457, *p* = 0.024; Table 3).

### 3.4. Full Path Predictors in Structural Model

Although overall model fit was not found to be appropriate, there were several paths which significantly influenced the intention to make appropriate concussion-related decisions (Figure 2, Table 3). Quality (precursor) significantly influenced perceived behavioral control (mediator; β = 0.122, *p* = 0.001) which influenced the intention to make an appropriate decision despite pressure (outcome; β = 0.118, *p* = 0.001). Quality also significantly influenced communication and collaboration practices (β = 0.082, *p* < 0.001) which influenced both the intention to make decisions despite pressure (β = 0.212, *p* = 0.044) and situation (β = 0.290, *p* = 0.016). Other significant paths did not go all the way through the proposed path (precursor → mediator → outcome) and instead only influenced one particular aspect (Figure 2).

## 4. Discussion

There were two types of proposed influential paths over concussion-related decision-making: first, partial paths (precursors → mediators; mediators → outcomes); and second, full paths (precursors → mediators → outcomes). The study data indicate that the IBM is likely not the most appropriate model for explaining the relationship between various factors and an individuals’ intention to make appropriate concussion-related decisions due to greater complexity of decision-making overall. Although the model in its entirety may not fully explain the paths of influence on intention, several individual factors had significant effects on the different path types. This may be in part due to the factors being more complex in nature and relationship than originally hypothesized. Although not perfect, these partial and full paths are still influential paths over secondary concussion prevention and therefore have strong clinical utility for sports medicine clinicians toward the reduction of further harm as they are modifiable and influence appropriate decision-making intentions.

Concussions are often complex to diagnose due to a lack of definitive diagnostic assessment tools (such as imaging) and reliance on self-report of symptoms [25,26,27,28,29,30]. As such, clinicians often have less confidence in decision-making around the injury. A primary driver of decreased confidence may be that nearly two-thirds of clinicians have felt pressure to expedite the return of a concussed athlete [11], as well as the heightened scrutiny and media attention placed upon these decisions, particularly when adverse outcomes occur [12]. A lack of definitive diagnostic test, pressure-filled decision-making, and high levels of attention placed upon the decisions indicate why certain factors evaluated in the current study were less important toward intentions including: quantity of educational focus on healthcare communication, knowledge, perceived norms, and salience. Additionally, the lack of statistically significant pathways found in our study may be due to limited construct variability; however, skewedness is frequent in studies including knowledge, attitudes, and perceived norms where certain values are more desirable [31,32,33].

Our results suggest that ATs’ communication practices directly influence intentions to make appropriate decisions. In ATs with fewer communicative relationships and communication time points, intentions to make an appropriate concussion-related decision were less endorsed. Therefore, ATs need to know how to communicate and collaborate with different stakeholders dependent on setting, appreciate the complexity of the decision as well as the gravity of the decision, be confident in the decision that they are making, and ultimately keep patient health as their primary goal. The inability to effectively communicate and collaborate may lead to increased stress, burnout, and interpersonal conflict, compromising the ability to effectively make and implement appropriate medical decisions [34]. Without quality education regarding communication and collaboration with all necessary stakeholders, communication and collaboration practices may suffer [35,36,37], impacting the ATs ability to make effective and efficient decisions in the moment, thereby impacting secondary concussion prevention and overall patient care. Stakeholders include other healthcare professionals as well as school personnel and families. Previous research and education have primarily focused on the physician-patient dyad without incorporating information regarding how to work with families and school personnel [38].

In recent years, concussion research has focused on and supported the use of a multidisciplinary injury management team [3,10]; however, multidisciplinary teams require strong efforts from all parties regarding communication and collaboration [39]. Using a team approach also may affect the ATs’ perceived behavioral control, or ability to do what they need to do in a particular situation. Our results suggest that Ats’ intentions are strongly influenced by perceived behavioral control, meaning a team approach can improve athlete care through improved decision-making practices. In order to optimize patient care and outcomes, the injury management team must have predetermined roles and responsibilities that all parties understand which has previously been an issue for ATs leading to increased pressure for decision-making [40,41,42,43]. The attempt to meet expectations of administrators, coaches, and athletes, as well as others, simultaneously create a unique challenge for ATs as they are frequently treated as an intermediary between stakeholders. Stress and pressure are heightened by competing obligations and expectations placed upon the AT which may impact their ability to perform their job in the most appropriate manner [44,45].

Although the model in its entirety may not be clinically actionable, the individual components highlight strong points of context for future educational initiatives for ATs and potentially other healthcare providers. As such, communication/collaboration initiatives should be a strong focus in current and future education of athletic training students to best prepare them to make appropriate clinical decisions that affect all levels of the prevention framework. Additionally, interventions for continuing education should be developed for currently ATs to strengthen their ability to communicate and collaborate, optimizing patient care. By improving communication and collaboration and utilizing a team approach, perceived behavioral control may be improved, thereby improving secondary concussion prevention efforts through appropriate decision-making. Lastly, these strategies should be researched and potentially adopted with decision-making as many injuries and pathologies in athletics are often complex and multidisciplinary in nature. Future research should also incorporate the experiences of how sport type and Name, Image, and Likeness (NIL) deals impact the pressure athletic trainers face compared to their professional setting counterparts. Additionally, future research should examine strategies to mitigate the pressure associated with decision-making to promote optimal athlete health.

### Limitations

These participants were solicited via a list provided by the NATA; therefore, if an AT was not a member of the NATA, they did not receive the invitation to participate. Most certified ATs are affiliated with the NATA and have clear lines for gathering possible research participant information. Therefore, the research team felt it was the most appropriate option. Additionally, participants may have self-selected to participate only due to pre-existing interest in the topic of study, although the study description was relatively vague preventing concern. Furthermore, participants may not have felt comfortable answering all survey questions honestly or attempted to conform to what they felt was the most appropriate response; however, due to the diversity of responses regarding influential factors, we feel this was not a substantial issue. In terms of participant demographics, the participants within this study were relatively early in their careers. It is possible that athletic trainers with more years of experience would have responded to the survey questions differently; however, we felt it was important to understand early career professionals as they had more actionable information tied to current educational standards. There was also a high response rate from sports that are less commonly covered by ATs. This is likely a by-product of the inclusion of high schools and smaller colleges with geographic prioritization of winter sports. In these instances, ATs are not specifically assigned a singular sport but are responsible for all sports within that school, leading to high numbers in less common sports. This may reduce the generalizability for ATs who are only responsible for a singular sport. Lastly, there is a substantial portion of missing data; however, most of these individuals dropped out within the first 7% of questions as opposed to enduring response fatigue. In addition, mean imputation was applied to 27.6% of cases which may result in over-estimation of actual values due to variable skewedness; yet, constructs for the path analysis did not significantly differ from individuals with full survey completion (*n* = 413). ATs have busy and unpredictable schedules; this study highlights a novel concept in athletic training literature that requires further investigation.

## 5. Conclusions

We hypothesized that several types of factors (educational and demographic precursors, theory-based factors, knowledge, salience, and communication/collaboration) would impact the intentions of ATs to make appropriate concussion-related decisions. Although found to not provide optimal model fit, results did show significant effects on the intentions to make appropriate decisions primarily focusing on communication and collaboration and perceived behavioral control. The inability to effectively communicate may increase stress and conflict relating to the perceived ability to perform job duties [34]. As such, it is important to establish and utilize a team approach focusing on the inclusion of interprofessional interactions between coaches, athletes, ATs, team physicians, and school personnel. By establishing a pre-determined team, communication and collaboration may be improved when an injury does occur due to the solidified foundation of understanding, thereby reducing pressure on the AT [25]. Reduced pressure allows for better initiation of secondary concussion prevention through appropriate action by a healthcare professional, such as an AT, to reduce the negative consequences of injury.

## Figures and Tables

**Table 1:** Participant demographics.

Variables	*n* (%)
Race * (*n* = 512)	
White	464 (90.6%)
Black	18 (3.5%)
American Indian/Alaska Native	7 (1.4%)
Asian	16 (3.1%)
Native Hawaiian/Pacific Islander	5 (1.0%)
Hispanic/Latino	37 (7.2%)
Unsure	1 (0.2%)
Other	1 (0.2%)
Gender (*n* = 512)	
Male	133 (26.0%)
Female	377 (73.6%)
Other	2 (0.4%)
Education (*n* = 512)	
Bachelors	190 (37.1%)
Masters	313 (61.1%)
Doctoral (PhD, EdD)	1 (0.2%)
Clinical Doctorate (DAT, DPT)	7 (1.4%)
Other	1 (0.2%)
Setting (*n* = 564)	
High School—Public	200 (35.5%)
High School—Private	42 (7.5%)
College—Division I	138 (24.5%)
College—Division II	51 (9.0%)
College—Division III	52 (9.2%)
College—NAIA	19 (3.4%)
College—Junior or Community	11 (2.0%)
Professional Sports	2 (0.4%)
Other	49 (8.7%)
Job Title (*n* = 508)	
Head AT	155 (30.5%)
Associate AT	17 (3.4%)
Assistant AT	148 (29.1%)
Outreach AT	59 (11.6%)
Graduate Assistant AT	107 (21.1%)
Other	22 (4.3%)
Reporting Structure (*n* = 504)	
Athletic Department	372 (73.8%)
Campus Health Services (non-athletic department)	34 (6.8%)
Medical School	9 (1.8%)
Other	88 (17.7%)
Current Sport Responsibilities * (*n* = 507)	
Baseball	271 (53.5%)
Men’s Basketball	283 (55.8%)
Women’s Basketball	285 (56.2%)
Bowling	39 (7.7%)
Cheerleading	285 (56.2%)
Men’s Cross Country	296 (58.4%)
Women’s Cross Country	62 (12.2%)
Men’s Diving	70 (13.8%)
Women’s Diving	1 (0.2%)
Men’s Fencing	1 (0.2%)
Women’s Fencing	234 (46.2%)
Men’s Field Event (Track)	239 (47.1%)
Women’s Field Event (Track)	64 (12.6%)
Field Hockey	287 (56.6%)
Football	190 (37.5%)
Men’s Golf	163 (32.2%)
Women’s Golf	5 (1.0%)
Men’s Gymnastics	35 (6.9%)
Women’s Gymnastics	42 (8.3%)
Men’s Ice Hockey	22 (4.3%)
Women’s Ice Hockey	104 (20.5%)
Men’s Lacrosse	107 (21.1%)
Women’s Lacrosse	13 (2.6%)
Performing Arts	11 (2.2%)
Rifle	19 (2.8%)
Men’s Rowing/Crew	9 (1.8%)
Women’s Rowing/Crew	9 (1.8%)
Men’s Skiing	252 (49.7%)
Women’s Skiing	270 (53.3%)
Men’s Soccer	263 (51.9%)
Women’s Soccer	140 (27.6%)
Softball	158 (31.2%)
Men’s Swimming	190 (37.5%)
Women’s Swimming	209 (41.2%)
Men’s Tennis	66 (13.0%)
Women’s Tennis	279 (55.0%)
Men’s Volleyball	21 (4.1%)
Women’s Volleyball	24 (4.7%)
Men’s Water Polo	182 (35.9%)
Women’s Water Polo	47 (9.3%)
Wrestling	207 (40.8%)
Other	14 (2.8%)

* Participants were allowed to select more than one response.

**Table 2:** Certified athletic trainer descriptives by construct.

Constructs	n	Median	IQR	Participant Range	Possible Range *
Quality of Educational Focus on Healthcare Communication	649	10.0	8.0–12.0	2.0–14.0	2.0–14.0
Quantity of Educational Focus on Healthcare Communication	587	33.0	20.0–60.0	2.0–1053.0	0.0–∞
Attitudes toward Concussion-Related Decision-Making	577	85.0	79.0–90.0	16.0–98.0	14.0–98.0
General	579	72.0	67.0–75.0	13.0–77.0	11.0–77.0
Complex	589	14.0	11.0–17.0	3.0–21.0	3.0–21.0
Perceived Norms toward Concussion-Related Decision-Making	630	3.0	2.5–3.0	−0.5–3.0	−3.0–3.0
Personal Agency toward Concussion-Related Decision-Making	574	29.0	27.0–32.0	20.0–35.0	5.0–35.0
Perceived Behavioral Control	591	15.0	13.0–18.0	4.0–21.0	3.0–21.0
Self-Efficacy	610	14.0	13.0–14.0	7.0–14.0	2.0–14.0
Knowledge of Concussion Symptoms and Consequences	582	82.0	79.0–85.0	54.0–98.0	25.0–100.0
Salience of Concussions	621	100.0	100.0–100.0	30.0–100.0	0.0–100.0
Communication/Collaboration Practices	701	3.0	2.0–3.0	0.0–3.0	0.0–3.0
Intention toward Concussion-Related Decision-Making	518	39.0	37.0–41.0	6.0–42.0	−45.0–45.0
Settings	541	6.0	6.0–6.0	−6.0–6.0	−6.0–6.0
Situations	523	21.0	19.0–21.0	0.0–21.0	−21.0–21.0
Pressures	525	15.0	15.0–15.0	0.0–15.0	−18.0–18.0

* Higher scores indicate better/safer/more positive constructs.

**Table 3:** Path analysis final model sources and paths of influence.

Variable	β (*p*-Value)						
	Perceived Behavioral Control	Complexity	General Attitudes	Communication/Collaboration	Intentions–Setting	Intentions–Pressure	Intentions–Situation
Quality	0.122 (*p* = 0.019)	0.187 (*p* = 0.008)	-	0.082 (*p* < 0.001)	-	-	-
Salience	-	-	-	-	0.013 (*p* = 0.004)	-	-
Perceived Behavioral Control	-	-	-	-	-	0.118 (*p* = 0.001)	-
Self-Efficacy	-	-	-	-	-	0.561 (*p* < 0.001)	0.805 (*p* < 0.001)
General Attitudes	-	-	-	-	-	0.033 (*p* = 0.012)	0.047 (*p* = 0.002)
Communication/Collaboration	-	-	-	-	-	0.212 (*p* = 0.044)	0.290 (*p* = 0.016)
Age	0.175 (*p* = 0.001)	-	-	-	-	-	-
Degree	-	-	1.748 (*p* = 0.021)	-	-	-	-
Setting	-	-	-	−0.317 (*p* < 0.001)	-	-	0.457 (*p* = 0.024)

Only values with statistical significance at alpha level 0.05 are displayed in the table. Model Fit Indices: RMSEA = 0.14, 95%CI: 0.13–0.16; CFI = 0.70. RMSEA, root mean square error of approximation; CFI, comparative fit index.

## Data Availability

The data presented in this study are available on request from the corresponding author. The data are not publicly available due to institutional review board data sharing requirements.

## Appendix A

**Table A1:** Survey Questions by Construct.

Overall Construct	Path Construct	Questions
Attitudes		Withholding an athlete from participation for a concussion (even if warranted) is:
	General	Weak (1)–Strong (7)
		Cruel (1)–Kind (7)
		Selfish (1)–Cooperative (7)
		Emotional (1)–Scientific (7)
		Risky (1)–Safe (7)
		Inefficient (1)–Efficient (7)
		Aimless (1)–Motivated (7)
		Bad (1)–Good (7)
		Unimportant (1)–Important (7)
		Foolish (1)–Wise (7)
		Unnecessary (1)–Necessary (7)
	Complexity	Uncomfortable (1)–Comfortable (7)
		Complicated (1)–Simple (7)
		Subjective (1)–Objective (7)
Perceived Norms	Not in Final Path	Most ATs make the appropriate decision to withhold an athlete for a concussion
		Most ATs approve of other ATs making the decision to withhold an athlete for a concussion
Personal Agency	Self-Efficacy	I am confident that I could respond to a concussion-related scenario (provided in survey) appropriately
		I am confident that I know when an athlete needs to be withheld for a concussion
	Perceived Behavioral Control	Withholding an athlete from participation for a concussion (even if warranted) is:
		Extremely difficult (1)–Extremely easy (7)
		Not under my control (1)–Under my control (7)
		Not influenced by others (1)–Influenced by others (7) ^a^
Salience	Salience	Please rank the level of priority placed upon making appropriate concussion-related decisions
Communication/Collaboration	Communication/Collaboration	How many times have you communicated/collaborated regarding making the decision to remove a concussed athlete?
		How prepared do you feel athletic training students are, in general, for autonomous decision-making upon graduation from a professional-level athletic training program?
		How prepared do you feel you were for autonomous decision-making upon graduation from a professional-level athletic training program?
Knowledge	Not in Final Path	I am confident that each of the following are symptoms of a possible concussion
		Things smell funny ^a^
		Things taste funny ^a^
		Problems remembering things
		Fuzzy vision
		Black eye ^a^
		Bleeding from ear ^a^
		Bleeding from mouth ^a^
		Headache
		Trouble sleeping
		Blacking out
		Stomach feels funny/hurts
		Numbness/tingling
		Weakness in your neck
		Chest pain ^a^
		Trouble understanding things
		Dizzy (feeling woozy)
		I am confident that each of the following are consequences of returning to play from a concussion too soon
		There are no consequences ^a^
		Skin rash ^a^
		More likely to suffer another
		Brain damage
		I am confident that each of the following are consequences of suffering multiple concussions
		There are no consequences ^a^
		Skin rash ^a^
		More likely to suffer another
		Brain damage
		Trouble remembering
Intentions		Intention to make appropriate concussion-related decision:
	Intentions (Setting)	On the sideline
		In the clinic
	Intentions (Situation)	During a game
		During a practice
		During the pre-season
		During the season
		During playoffs/championships
		During the off-season
	Intentions (Pressure)	Despite pressure from a coach
		Despite pressure from administration
		Despite pressure from a parent
		Despite pressure from an athlete
		Despite pressure from a fan

^a^ These items are reverse coded to reflect incorrect symptoms/consequences as negative impacts to the overall construct total.
